# Discovery and validation of serum glycoprotein biomarkers for high grade serous ovarian cancer

**DOI:** 10.1002/prca.202200114

**Published:** 2023-06-01

**Authors:** Mriga Dutt, Gunter Hartel, Renee S. Richards, Alok K. Shah, Ahmed Mohamed, Sophia Apostolidou, Aleksandra Gentry-Maharaj, Australian Ovarian Cancer Study Group, John D. Hooper, Lewis C. Perrin, Usha Menon, Michelle M Hill

**Affiliations:** 1 QIMR Berghofer Medical Research Institute, Brisbane, QLD, Australia 4006; 2 MRC Clinical Trials Unit, Institute of Clinical Trials and Methodology University College London, London, UK; 3 Mater Research Institute – The University of Queensland, Translational Research Institute, Woolloongabba, QLD 4102, Australia; 4 Mater Adult Hospital, South Brisbane, QLD 4101, Australia; 5 UQ Centre for Clinical Research, Faculty of Medicine, The University of Queensland, Brisbane, Australia 4006

**Keywords:** high grade serous ovarian cancer, lectin magnetic bead array (LeMBA), mass spectrometry, ovarian cancer screening, serum glycoprotein biomarker

## Abstract

**Purpose:**

This study aimed to identify serum glycoprotein biomarkers for early detection of high-grade serous ovarian cancer (HGSOC), the most common and aggressive histotype of ovarian cancer.

**Experimental design:**

The glycoproteomics pipeline lectin magnetic bead array (LeMBA)-mass spectrometry (MS) was used in age-matched case-control serum samples. Clinical samples collected at diagnosis were divided into discovery (n=30) and validation (n=98) sets. We also analysed a set of preclinical sera (n=30) collected prior to HGSOC diagnosis in the UK Collaborative Trial of Ovarian Cancer Screening.

**Results:**

A 7-lectin LeMBA-MS/MS discovery screen shortlisted 59 candidate proteins and 3 lectins. Validation analysis using 3-lectin LeMBA-multiple reaction monitoring (MRM) confirmed elevated A1AT, AACT, CO9, HPT and ITIH3 and reduced A2MG, ALS, IBP3 and PON1 glycoforms in HGSOC. The best performing multimarker signature had 87.7% area under the receiver operating curve, 90.7% specificity and 70.4% sensitivity for distinguishing HGSOC from benign and healthy groups. In the preclinical set, CO9, ITIH3 and A2MG glycoforms were altered in samples collected 11.1±5.1 months prior to HGSOC diagnosis, suggesting potential for early detection.

**Conclusions and clinical relevance:**

Our findings provide evidence of candidate early HGSOC serum glycoprotein biomarkers, laying the foundation for further study in larger cohorts.

## 1 Introduction

While ovarian cancer is only the third most common gynaecological cancer worldwide, it is the leading cause of gynaecological cancer mortality. One of the main factors for this high mortality rate is diagnosis at an advanced stage due to non-specific symptoms and the lack of effective predictive or diagnostic blood biomarkers. Late diagnosis is associated with high mortality. Only 29% of women with distant metastases survive five years, compared to 92% with localized disease [1]. However, the existing ovarian cancer diagnostic tests in clinical use, transvaginal ultrasound and serum CA125, do not have the sensitivity required for detecting the disease in early stage. Indeed, two large ovarian cancer screening trials using a combination of these modalities found no evidence of a reduction in disease-specific mortality on long-term follow-up [2, 3]. Furthermore, neither test is specific to cancer and both trials reported unnecessary surgery in women without cancer [4]. This has led to a concerted effort to discover biomarkers for early detection of ovarian cancer [5].

Cancer is associated with alterations in the glycosylation machinery and glycan structures on circulating proteins [6]. Several studies have shown that specific glycoforms of cancer biomarkers can improve specificity. For ovarian cancer, glycosylated forms of CA125 measured by microarray [7], lectin immunoassay [8] or glycosylation-specific antibodies [9] can significantly improve differential diagnosis. This suggests that a glycoform-specific glycoprotein biomarker panel may achieve the high specificity and sensitivity required for ovarian cancer screening. Glycomic and glycoproteomics studies on ovarian cancer serum and tissues have revealed differential abundance of several types of N-glycans in ovarian cancer, including fucose, sialic acid, high mannose types [10–15]. However, these potential biomarkers are yet to be clinically validated.

Here, we report on a study using lectin magnetic bead array (LeMBA)-coupled mass spectrometry platform [16] for ovarian cancer serum glycoprotein biomarker discovery and validation. LeMBA is a one-pot, high throughput glycoproteomics method with no need for abundant serum protein depletion, potentially increasing robustness of the biomarker development process as we previously reported for oesophageal adenocarcinoma [17, 18] and canine haemangiosarcoma [19]. It has not been previously used for glycoprotein biomarker studies in ovarian cancer. We have focused on the most common and aggressive histotype, high grade serous ovarian cancers (HGSOC), which accounts for ~70% of ovarian cancers and most of the disease-specific mortality [5]. Furthermore, for discovery of biomarkers with the potential for early detection, in addition to using samples collected at clinical diagnosis as is the norm, we also evaluated samples collected prior to ovarian cancer diagnosis from the multicentre randomised controlled trial, the United Kingdom Collaborative Trial of Ovarian Cancer Screening (UKCTOCS).

## 2 Materials and methods

### Study design

Case control studies were undertaken using serum samples from women with HGSOC patients (cases) and two age-matched groups (controls) – women with a benign ovarian neoplasm and healthy women using sample sets from three independent cohorts - (1) a clinical set of 30 serum samples from the United Kingdom Ovarian Population Study (UKOPS) collected from women at diagnosis of HGSOC (n=10), benign ovarian neoplasms (n=10) and healthy controls (n=10). (Table S1); (2) a pre-clinical set of 30 serum samples from the UKCTOCS trial [20] collected from women at a mean interval of 11.1±5.1 months prior to diagnosis of HGSOC (n=10), benign ovarian neoplasms (n=10) and healthy controls (n=10). (Table S1); and (3) a clinical set of 95 serum samples from the Australian Ovarian Cancer Study (AOCS) collected from women at diagnosis of HGSOC (n=39), benign ovarian neoplasms (n=28) and healthy controls (n=28). (Table S2)

**Figure 1:**
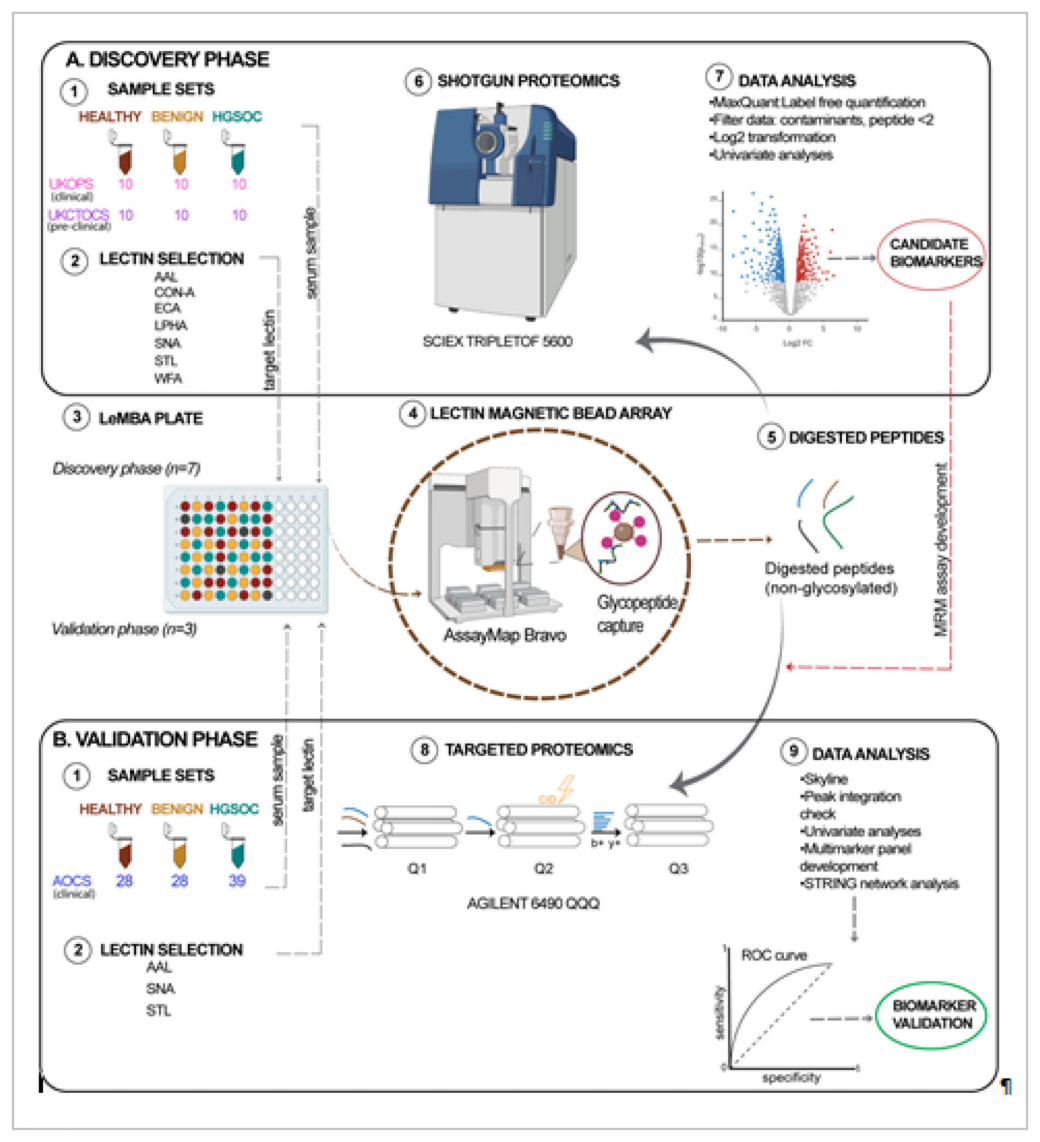
Biomarker study design. Discovery and validation of HGSOC biomarkers was conducted in two phases, starting from separate clinical cohorts (1) and sera collection (2). Lectin selection (3) was based on literature for discovery phase and the discovery results for the validation phase. Both phases use LeMBA (4), liquid handler-assisted pulldown (5) and on-bead digestion (6). Shotgun mass spectrometry was conducted for discovery phase (7) followed by discovery of candidates (8) for development of a targeted mass spectrometry assay (9) for validation phase. Both univariate and multivariate analyses were conducted for biomarker validation (10).

The discovery phase included the UKOPS clinical set and the UKCTOCS pre-clinical set. A shortlist of candidate proteins and lectins from the discovery phase was then validated using the independent clinical set from the AOCS (Figure 1). This study was approved by the QIMR Berghofer Medical Research Institute Research Ethics committee, and the East Midlands - Derby Research Ethics Committee in the UK. AOCS was approved by the Human Research Ethics Committees at the Peter MacCallum Cancer Centre, QIMR Berghofer Medical Research Institute, University of Melbourne and all participating hospitals.

### Biomarker Discovery Phase

LeMBA-MS was used for both discovery and validation phases (Figure 1). For discovery, seven lectins were selected from the literature [10, 11, 13, 15]: *Aleuria aurantia* (AAL), Concanavalin-A (Con-A), *Erythrina cristagalli* (ECA), *Phaseolus vulgaris* Leucoagglutinin (L-PHA), *Sambucus nigra* (SNA), *Solanum tuberosum* (STL) and *Wisteria floribunda* (WFA), which preferentially target glycoproteins with fucose (α1-3, α1-4, α1-6 linked), mannose (oligomannose and hybrid-type), galactose (β1-4-linked terminal), 2,6-branched tri-, tetraantennary complex-type N-glycan, sialic acid (α2-6-linked and Tn antigen), N-acetylglucosamine ((GlcNAcβ1-4)n (Chitin), oligosaccharide containing GlcNAc and MurNAc), and N-acetylgalactosamine (terminal), respectively. All chemicals and reagents were purchased from Sigma-Aldrich, USA unless stated otherwise.

#### Serum denaturation

Thawed serum samples were centrifuged at 16,000 g and 4°C for 15 min to remove cellular debris and the supernatant protein concentration was determined by BCA protein assay (Pierce™, Thermo Fischer). To minimise batch effects, bulk serum denaturation was performed for the entire project. An aliquot of each serum sample containing 800 μg of protein was diluted to 10 μg/μl in denaturation buffer (20 mM Tris-HCl pH 7.4, 1% v/v sodium dodecyl sulfate (SDS) and 5% v/v Triton X-100. The internal standard protein chicken ovalbumin was added to each serum sample at 10 pmol. Protein disulfide bonds were reduced by adding 20 mM dithiothreitol (Thermo Fisher, USA) to the samples and incubating at 37°C for 30 min. Following this, 100 mM iodoacetamide (Thermo Fisher, USA) was added to each sample and incubated at room temperature for 30 min in the dark to alkylate free thiol groups. The denatured serum samples were diluted 20 times in LeMBA binding buffer (20 mM Tris-HCl pH 7.4, 300 mM NaCl, 1 mM CaCl_2_, 1 mM MnCl_2_, 1% Triton, 1 unit protease inhibitor cocktail) to yield a final protein concentration of 0.5 μg/μl. Aliquots of 100 μl were transferred to microplates in a randomized layout in preparation for LeMBA. Prepared plates were sealed and frozen at -80°C until use.

#### Lectin magnetic bead pulldown and on-bead trypsin digest

LeMBA-MS with the selected seven lectins was performed as previously described [16, 18]. First, individual lectins were conjugated to MyOne™ tosyl-activated Dynabeads™ (Invitrogen, Australia) by incubating 50 μg of selected lectin (Vector Laboratories, USA) with 100 μl of Dynabeads™ at 37 °C for 24 h. The resulting lectin-bead conjugate was treated with 2% w/v glycine solution to reduce nonspecific binding, and further incubated at 37 °C for 16 h. The blocked beads were washed and diluted in lectin storage buffer (20 mM Tris pH 7.4, 150 mM NaCl, 1 mM CaCl_2_, 1 mM MnCl_2_, 0.5% Triton-X 100, 1 unit protease inhibitor cocktail).

Pulldown using the prepared lectin magnetic beads was performed on the AssayMAP Bravo liquid handler workstation (Agilent Technologies, USA) using one lectin per microplate. Briefly, 50 μl conjugated beads and 100 μl denatured serum was added to each well of a 96-well microtiter plate (Greiner, USA), and glycoprotein capture was performed at 4°C for 1 h under gentle shaking. Post incubation, the conjugated beads with the captured glycoproteins were washed seven times in 50 mM ammonium bicarbonate buffer with two microplate changes to minimise trace detergent, reducing and alkylating agent concentrations. The captured glycoproteins were digested with trypsin at 37°C for 18 h after adding 50 mM ammonium bicarbonate buffer and 1 μg of sequencing grade porcine trypsin (Promega, Australia) to each well. The plate was sealed for enzyme digestion. The trypsin was inactivated with 1% v/v formic acid (FA; Merck, USA) and the digested peptides were collected, dried down in a vacuum concentrator, sealed and stored at -80°C until use.

#### Data dependent acquisition mass spectrometry

Shotgun proteomics using data-dependent acquisition was performed on a SCIEX 5600 TripleTOF 5600+ mass spectrometer (SCIEX, USA) coupled to a Shimadzu LC-20AD Prominence nano liquid chromatography system (Shimadzu, Japan). All solvents and reagents were of mass spectrometry grade (Thermo Fisher, USA). The mass spectrometer was controlled using Analyst 1.7 software (SCIEX, USA). Digested peptides were resuspended in 0.1% v/v FA and injected onto a Protecol C18 analytical column (200 Å, 3 μm, 150 mm x 150 μm, Trajan Scientific, Australia) connected to a Protecol guard column (Polar 120 Å, 3 μm, 10 mm x 300 μm, Trojan Scientific, Australia) and the sample injection order was randomised in the worklist. Column compartment was maintained at 45°C. The peptides were eluted using mobile phase A (0.1% v/v FA) over the specified gradient of mobile phase B (95% acetonitrile, 5% v/v water, 0.1% v/v FA) for 60 min at a flow rate of 1.2 μl/min (5% B at 3 min; 30% B at 37 min; 50% B at 45 min; 100% B at 47min; 100% B at 51 min; 5% B at 53 min until end of run). The nanospray ion source was set as follows: ion source gas 1 = 35 psi, curtain gas = 30 psi, ion spray floating voltage = 2400 V and interface heater temperature = 180°C. The ion optics parameters were set as declustering potential = 100 V and collision energy (survey scan) = 10 V. Data acquisition was performed using the information dependent acquisition (IDA) and the top 30 precursors from each survey scan were selected for fragmentation. The MS1 spectra was acquired in positive polarity within the mass range = m/z 350 — 1250 Da, with the accumulation time of 250 ms. The precursor selection mass window in the quadrupole was set to unit resolution (m/z 0.7 window). The MS/MS spectra were acquired using collision induced dissociation (CID) within the mass range = m/z 100 — 1500 Da with the following parameters: charge states +2 to +5, accumulation time= 100 ms, fragmentation threshold = 150 cps, dynamic exclusion =15 s and d collision energy voltage was set as rolling collision energy with a collision energy spread of 3.

The acquired raw ion spectra for each lectin batch were searched against the reviewed UniProt human proteome database (20,365 proteins, accession date 01/01/2020) using MaxQuant software, v.1.6.6.0 [21]. The MaxQuant contaminant database (247 entries) was also searched to identify contaminants such as keratin. MaxQuant parameters were set as follows: Digestion = trypsin, with 2 missed cleavages; fixed modification was set to cysteine carbamidomethylation; variable modifications were set as methionine oxidation and N-terminal acetylation; label free quantification (LFQ) was enabled with minimum ratio count set to 2; unique and razor peptides were used for protein identification; match between runs was set as TRUE; and false discovery rate (FDR) for protein and peptide identification was set at 0.01. AB Sciex Q-TOF was set as instrument type using default settings. First search peptide tolerance was set to 0.07 Da and 0.06 Da for the main search. MS/MS tolerance was set at 40 ppm. The search results were imported into R software v1.4.1103 (www.R-project.org) for further data processing and statistical analyses.

#### Mass spectrometry data processing and statistical analysis

The generated protein list for each lectin batch was filtered to remove contaminants, reverse identified protein IDs, proteins with < 2 peptide IDs and score < 5. Proteins which were missing in < 25% of all samples were considered missing at random and imputed using localized least square regression (llsimpute) [22]. Proteins missing in > 25% were imputed with the minimum detected value (values drawn randomly from a normal distribution centered at sample minimum and with SD estimated from non-missing proteins). Quantitative analysis was conducted at the protein level using the summed intensity of all peptides mapped to each protein. Log2 transformed data was analyzed using the R limma package [23] and Student’s T-test. As candidates from the discovery phase will be further confirmed by targeted mass spectrometry, and application of false discovery rate to the dataset yielded very few significant differences, we shortlisted candidates based on non-adjusted p-values. Differentially abundant proteins were visualised by volcano plots, using the criteria p-value <0.05 and Log_2_ fold change >1, and the nomenclature “lectin-UniProt entry name”. All graphical output has been generated using R or GraphPad Prism v9 (San Diego, USA) and figures prepared using Illustrator v26.3.1 (Adobe Inc, USA) and Biorender (www.biorender.com).

### Biomarker validation phase

#### Multiple reaction monitoring assay development

A custom multiple reaction monitoring (MRM) assay was developed for the list of protein biomarker candidates discovered from UKCTOCS and UKOPS clinical sets, after manually removing immunoglobulins from the list. An initial transition list was selected by matching to a spectral library generated from *in silico* trypsin digest of the discovery phase raw spectral files (n=420) in Skyline v 21.1.0.278 (http://skyline.maccosslab.org/), using human proteome as background proteome. For each candidate biomarker protein, a minimum of ten peptides were selected, each consisting of at least six transitions (b and y ions). Additionally, the “Unique Peptides” parameter in Skyline was applied to check for peptide uniqueness to a single protein.

For MRM method optimisation, digested peptide samples from all the lectin batches and across both discovery sets were pooled into a single sample. To monitor retention time across all runs, three stable isotope standard (SIS) peptides (VTSIQDWVQ**K**, NLAVSQ**V**VHK, LSPIYNL**V**PVK) were added. The final dynamic MRM method consisted of 60 proteins (59 candidate biomarker proteins + 1 chicken ovalbumin protein), 176 peptides (170 candidate peptides + 3 SIS peptides + 3 chicken ovalbumin peptides) and 860 transitions with a delta retention time of 1 min.

#### LeMBA-MRM-MS

Candidate biomarker validation was performed on the independent clinical sample set from AOCS. Based on the discovery phase results, AAL, SNA and STL lectins were selected for the validation phase and the serum samples were subjected to the LeMBA workflow as described in the discovery phase. Prior to mass spectrometry injection, the above three SIS peptides were spiked-in to the samples for monitoring retention time stability across runs. MRM-MS was performed on an Agilent 6490 triple quadrupole mass spectrometer coupled to an Agilent 1290 Infinity UHPLC system, equipped with an Agilent jet stream + ESI source. The mass spectrometer was controlled by MassHunter software (Agilent Technologies, USA). Digested peptides (10 μl, 20 μg) were injected onto a reverse phase AdvanceBio Peptide Mapping analytical column (150 X 2.1 mm i.d., 2.7 μm, part number 653750-902, Agilent Technologies, USA) connected to a 5 mm long guard column and the sample injection order was randomised in the worklist. The column compartment was maintained at 50 °C. The peptides were eluted using mobile phase A (0.1% v/v FA) over the specified gradient of mobile phase B (100% acetonitrile, 0.1% v/v FA) for 35 min at a flow rate of 0.4 ml/min (3% B at 0 min; 30% B at 20 min; 40% B at 24 min; 95% B at 24.5 min; 95% B at 28.5 min and 3% B at 29 min until end of run). The mass spectrometer operated in positive ion mode and the source parameters were set as 150 V high pressure RF, 60 V low pressure RF, 4000 V capillary voltage, 300 V nozzle voltage, 30 psi nebulizer gas flow, 15 L/min drying gas flow at a temperature of 150 °C, 11 L/min sheath gas flow at a temperature of 250 °C and 200 V delta EMV. The quadrupole was set at unit resolution (0.7 Da full width at half maximum in the first quadrupole (Q1) and the third quadrupole (Q3)), fragmentor at 380 V and cell accelerator voltage at 4 V.

Due to a mass spectrometer software failure, data for the first batch of AAL-pulldown samples were not saved. The entire plate had to be re-run using remaining sample volume, and 25 samples were noted to have lower remaining volume. In addition, an injection problem was noted for one SNA-pulldown sample.

#### Data analysis

The data analysis for each lectin MRM-MS was performed independently with no comparisons performed across the three lectins. MRM-MS raw data for the three lectins were exported to Skyline v 21.1.0.278 (downloaded January 2022, http://skyline.maccosslab.org/) to manually check for correct peak integration and the peak area of each measured transition was exported and further analysed in R (v1.4.1103). For each measured peptide in each sample, the transition peak areas were summed and then Log_2_ transformed.

Data quality control was conducted using peak area for the three internal standard chicken ovalbumin peptides (Figure S1) and the three spiked-in SIS peptides (Figure S2). While most chicken ovalbumin peptides were consistent, the 25 AAL and 1 SNA samples with noted aberrations at the mass spectrometry step showed larger variability, and were removed as outliers (Figure S1, S2). After outlier removal, the calculated %CV for all peptide standards was less than 10% (except peptide AAL-VASMASEK, 11.3%) (Table S3) and the peak area distribution of all samples remaining in the analysis exhibited a normal data distribution (Figure S3). As the dataset has low %CV, we decided normalisation is not required. Student’s t -test was performed between the case control groups and false discovery rate using Benjamini-Hochberg method was applied to identify significantly differing proteins at p-adjusted-value <0.05. All graphical output has been generated using R or GraphPad Prism and figures prepared using Adobe Illustrator and Biorender.

#### Multi-marker panel development

Generalized regression with binomial distribution and lasso estimation was used to develop multi-marker panels using JMP Pro version 16.2.0 (JMP Pro Inc., Carey, NC, USA). Performance of the multi-marker panels were assessed using leave-one-out cross-validation, where area under the receiver operating curve, specificity, sensitivity was calculated on the left-out observations. The number of times each parameter (peptide) was chosen in the cross-validation models is presented as an indication of the relative importance of the markers for prediction. All models were also run using peptides standardised by subtraction of the mean of the three internal standard chicken ovalbumin peptides, but are not reported as they yielded comparable results. Inclusion of the 25 AAL outliers also was tested and lead to similar albeit slightly worse prediction models.

## 3 Results

### Discovery of candidate serum glycoprotein biomarkers

**Figure 2:**
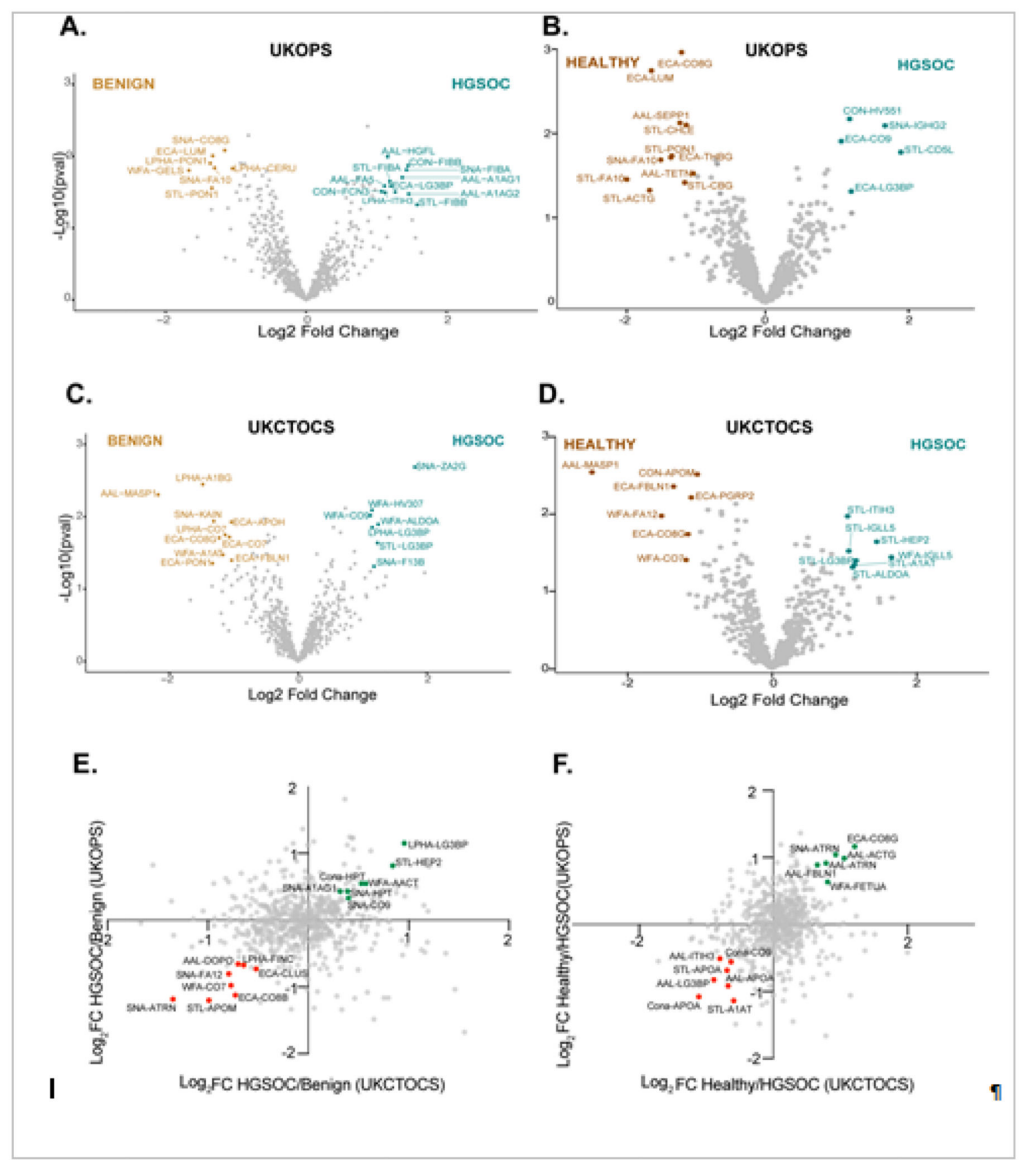
Biomarker discovery data. Volcano and two-way scatter plots visualising the differentially abundant proteins and correlated proteins respectively, between the benign and HGSOC (A, C, E) and healthy and HGSOC (B, D, F) clinical comparisons for UKOPS and UKCTOCS sample sets. The volcano plots highlight all differential glycoproteins according to the criteria p<0.05, Log_2_ Fold Change >1. The scatter plots highlight select glycoproteins (Log_2_ Fold Change >0.5) that are upregulated (green dots) and downregulated (red dots) in both sample sets. All candidates are indicated using the nomenclature “lectin-Uniprot entry name”.

In the UKOPS discovery set, we found 15 and 16 differentially abundant proteins when HGSOC samples were compared to benign and healthy samples, respectively (Figure 2, Table 1, Table S4). Compared to benign samples, UKOPS HGSOC samples exhibited an increase in AAL-A1AG1 (alpha-1-acid-glycoprotein 1), AAL-A1AG2 (alpha-1-acid-glycoprotein-2), AAL-FA5 (coagulation factor V), AAL-HGF (hepatocyte growth factor), STL-FIBA (fibrinogen alpha chain), AAL-FIBB (fibrinogen beta chain), LPHA-ITIH3 (inter-alpha-trypsin-inhibitor heavy chain 3), as well as decreased SNA-CO8G (complement component C8 gamma chain), SNA-FA10 (coagulation factor X), LPHA-PON1 serum paraoxonase/arylesterase 1) and STL-PON1 (Figure 2A). When compared to healthy samples, the UKOPS HGSOC samples exhibited an increase in ECA-CO9 (complement component C9) and ECA-LG3BP (galectin-3-binding protein), and a reduction of ECA-C8B (complement component C8B), ECA-CO8G, ECA-LUM (lumican) and ECA-THBG (thyroxine-binding globulin), STL-ACTG (actin), STL-CBG (corticosteroid-binding globulin, STL-CHLE (cholinesterase) and STL-PON1 (Figure 2B).

In the UKCTOCS discovery set, we found 14 and 12 differentially abundant proteins in HGSOC samples when compared to benign and healthy samples respectively (Figure 2, Table 1, Table S5). Compared to benign samples, UKCTOCS HGSOC samples showed an increase in WFA-ALDOA (fructose bisphosphates aldolase A), WFA-CO9 and WFA-HV307 (immunoglobulin heavy-variable 3-7), SNA-F13B (coagulation factor XIII B chain) and SNA-ZA2G (zinc-alpha-2-glycoprotein), as well as reduction in AAL-MASP1 (mannan-binding lectin serine protease 1), ECA-APOH (beta-2 glycoprotein-1), ECA-CO7 (complement component C7), ECA-CO8G, ECA-FBLN1 (fibulin-1) and ECA-PON1 d (Figure 2C). On the other hand, when compared to healthy samples, HGSOC samples showed increased STL-A1AT (alpha-1-antitrypsin), STL-HEP2 (heparin cofactor 2), STL-ITIH3, STL-IGLL5 (immunoglobulin lambda like growth factor), STL-LG3BP, and reduced AAL-MASP1, ECA-CO8G, ECA-FBLN1 and ECA-PGRP2 (peptidoglycan recognition protein-2), WFA-CO7 and WFA-FA12 (coagulation factor XII)(Figure 2D).

Additionally, there was an overlap of a subset of candidates that displayed the same expression trend in both the sample sets such as LPHA-LG3BP, STL-HEP2 and SNA-CO9 in the HGSOC versus benign comparison (Figure 2E) and ECA-C08G, SNA-ATRN and STL-A1AT in the healthy versus HGSOC comparison (Figure 2F).

### Validation of candidate biomarkers

Three lectins (AAL, SNA and STL) with the largest number of candidate proteins discovered in both UKOPS and UKCTOCS sets (Table 1), were selected for validation in the independent clinical AOCS cohort. The list of protein candidates discovered from UKOPS and UKCTOCS were combined to generate a list of 44 proteins, which fell short of the target number of ~60 candidate proteins that we previously used for biomarker validation [17, 18]. In order to assess additional candidates which may be just outside of the p<0.05 cut-off, we expanded the selection threshold to p-value <0.1 and removed Log_2_ fold change filtering. This resulted in an initial list of 102 proteins which was filtered down to a final MRM target list of 59 candidates based on suitability of protein tryptic peptides for MRM. The developed custom MRM assay measured 170 peptides from the 59 candidate proteins, with at least 3 peptides per protein and 4-5 transitions per peptide. The full MRM-MS data are provided in Table S6.

**Figure 3:**
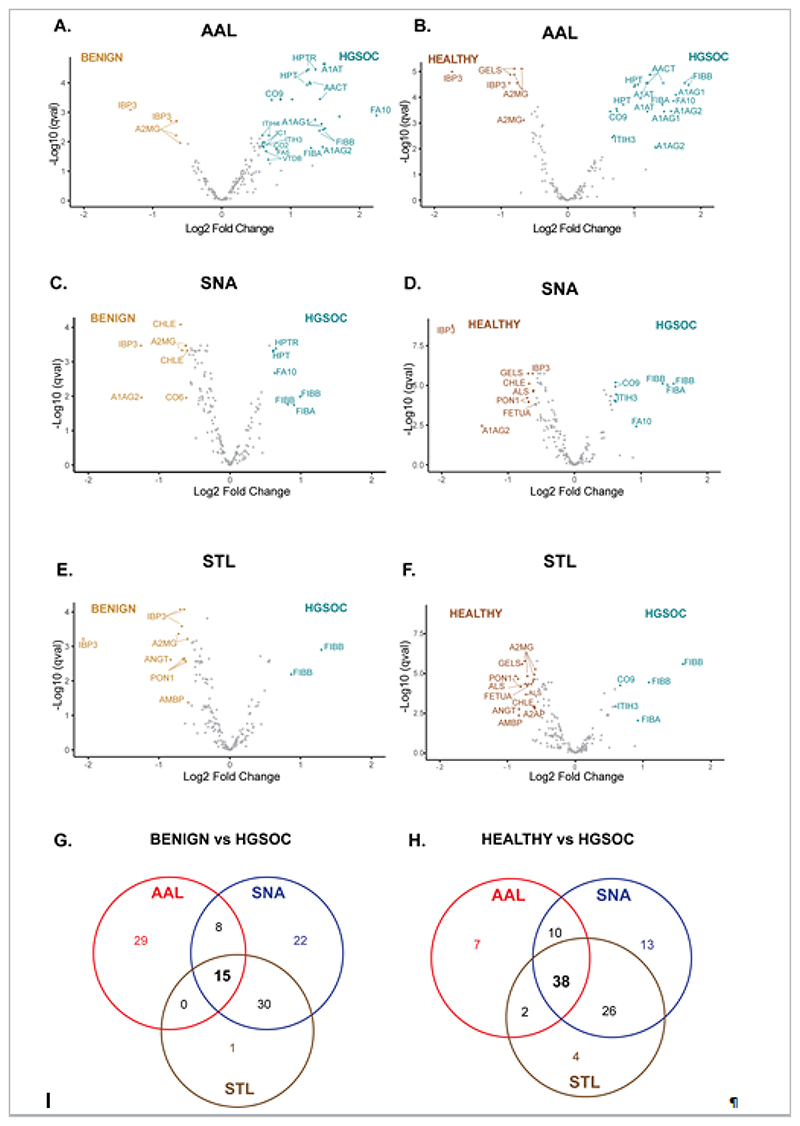
Biomarker validation data. LeMBA-MRM data were analyzed for differentially abundant peptides between benign and HGSOC (A, C, E, G), and healthy and HGSOC (B, D, F, H) for AAL (A, B), SNA (C, D), and STL (E, F) lectins respectively. Each dot in the volcano plot indicates a peptide, labelled only by the corresponding UniProt ID for the protein for visualization. The overlap between candidates for each lectin is shown in G, and H.

Univariate analysis for HGSOC versus benign and HGSOC versus healthy samples was conducted at the peptide level on each lectin dataset, with significance cut-offs set at adjusted p-value <0.05 and log2 fold change >0.5 (Table S7). Overall, for the HGSOC versus benign comparison, we found 53 (AAL), 80 (SNA) and 49 (STL) peptides with an overlap of 15 common peptides that were mapped to 7 proteins (Figure 3A, C, E, G). Likewise, for the HGSOC vs healthy comparison, we found 58 (AAL), 88 (SNA) and 74 (STL) peptides, with an overlap of 38 peptides that were mapped to 18 proteins (Figure 3B, D, F, H). To summarise the peptide differential expression data into HGSOC glycoprotein biomarkers, we next looked for consistency in the peptide differential expression. Each glycoprotein candidate was measured by 3 or more non-glycosylated peptides, but not all peptides showed significance or consistent direction of change, possibly related to proteoforms or protein cleavage. Filtering for consistent direction of change (up/down) across all measured peptides revealed 21-31% of proteins had all consistent peptides, with 5 proteins elevated in HGSOC (A1AT, AACT, CO9, HPT and ITIH3), and 4 down-regulated proteins (A2MG, ALS, IBP3 and PON1) across the three lectins (Table 2). The validated glycoproteins had diverse lectin-binding affinities, from binding to all three lectins (CO9, A2MG) to a single lectin (AAL-HPT, STL-PON1) (Table 2).

We used STRING v 11.5 to investigate the interactions between the nine validated biomarker proteins. The developed protein-protein interaction (PPI) network had 16 edges (expected 0), and significantly more interactions than expected (PPI enrichment p-value of <1 x 10^-16^) (Figure S4A). Functional enrichment analyses revealed significant enrichments in Gene Ontology Cellular Component terms Blood microparticle (5 out of 115 genes, FDR 1.67x10^-6^), extracellular exosome (8 out of 2099 genes, FDR 8.21 1.67x10^-5^), insulin-like growth factor ternary complex (2 out of 4 genes, FDR 0.0048), amongst others, as well as KEGG pathway, Complement and coagulation cascades (3 out of 82 genes, FDR 0.0022) (full analysis in Table S8).

### Development of multi-marker signature for HGSOC

The receiver operating curve (AUC), specificity and sensitivity of the developed multi-peptide models are detailed in Table 3, Table S9. All four models performed similarly with the AAL signature having the highest AUC (87.5%), sensitivity (70.4%) and specificity (90.7%). To further inspect the stable peptides for each of the models, we filtered peptides chosen in at least 50% of the cross-validation runs (Table 3). This analysis revealed several interesting observations. The IBP3 peptide ALAQCAPPPAVCAELVR was always selected for each lectin signature, indicating strong predictive value for HGSOC. Two peptides were highly stable for SNA, STL and the combined signatures, namely, A2MG_NEDSLVFVQTDK and CHLE_NIAAFGGNPK. For the combined signature, both SNA and STL binding IBP3_ALAQCAPPPAVCAELVR were selected with high stability (100% and 91.6%, respectively). Strikingly, the stable peptides in the combined signature comprised 2 SNA and 5 STL peptides, with no AAL peptides. PPI analysis again revealed significant interactions with enrichment value of 1.29 x 10^-7^ (Figure S4B), and enrichment of the GO Cellular Component term blood microparticle (4 out of 115 genes, FDR 3.28 x 10^-5^), as well as the KEGG pathway component and coagulation cascades (4 out of 82 genes, FDR 1.74 x 10^-6^). Additionally, the GO biological process blood coagulation, fibrin clot formation was also highly enriched (4 out of 26 genes, FDR 8.62x 10^-7^, Table S8).

### Evaluation of validated biomarker candidates for early HGSOC detections

**Figure 4:**
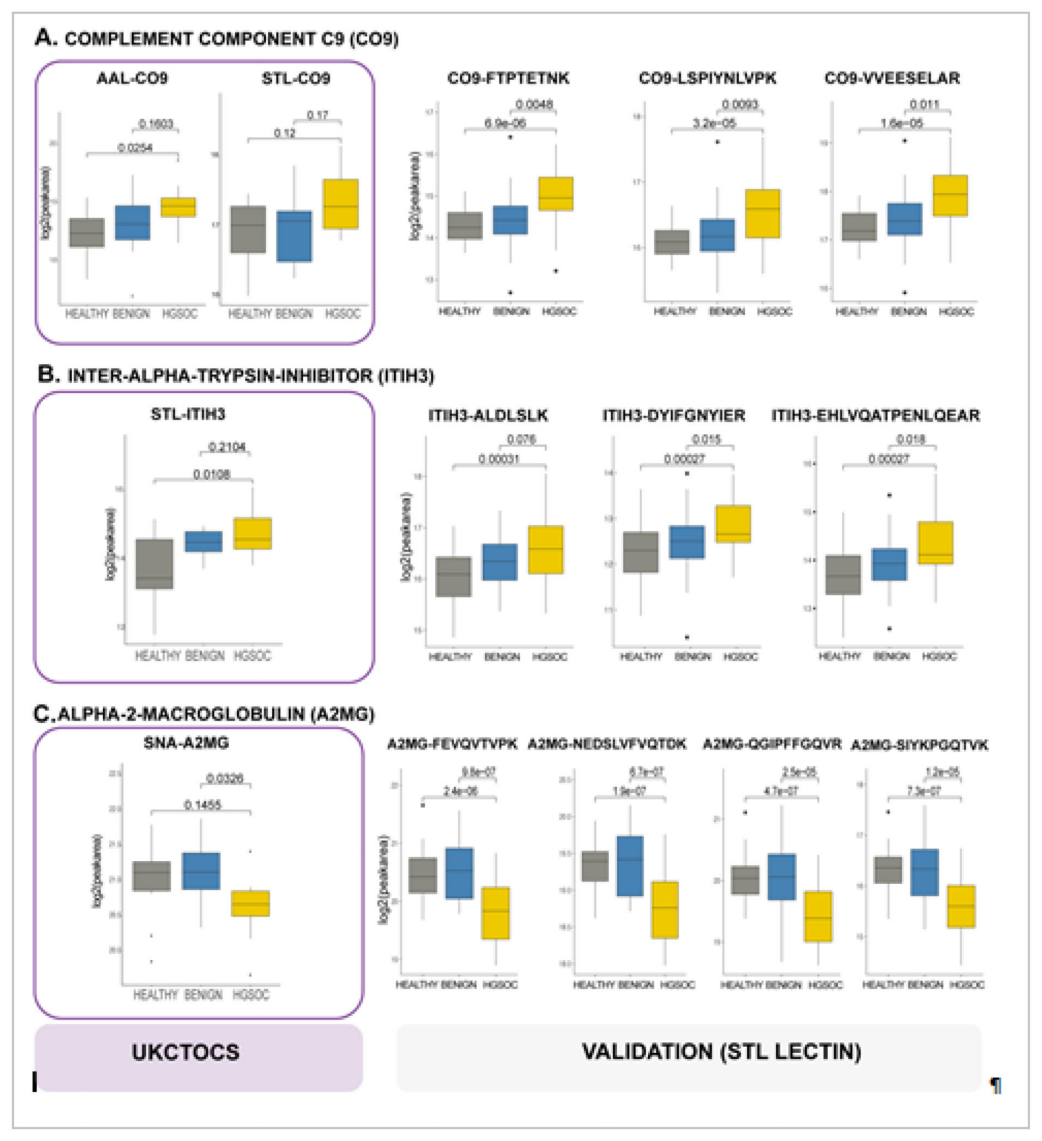
Potential HGSOC early detection biomarkers. Comparison of glycoprotein biomarker data between UKCTOCS discovery case control set (left panel, LeMBA-DDA-MS protein level data), and STL-pulldown validation case control set (right panel, LeMBA-MRM-MS, peptide level data) for (A) CO9, (B) ITIH3, and (C) A2MG. For comparison, the unadjusted p-values are shown for both data sets.

To determine if any of the nine validated univariate protein biomarkers were altered in the pre-clinical samples, we re-examined the UKCTOCS discovery LeMBA-DDA-MS data set for the nine proteins (Table 2). Three proteins, namely CO9, ITIH3 and A2MG were significantly altered in the UKCTOCS samples. Figure 4 illustrates the comparative data from discovery UKCTOCS (protein level) and validation (peptide level) phases. AAL-CO9 (Figure 4A) and STL-ITIH3 (Figure 4B) were significantly higher in HGSOC group compared to benign and healthy groups in the UKCTOCS set, while SNA-A2MG was lower in HGSOC group (Figure 4C). STL-CO9 was also elevated in HGSOC versus other groups in the UKCTOCS set, albeit not statistically significant (Figure 4A), while STL-A2MG was not detected in the UKCTOCS or UKOPS datasets likely due to the lower sensitivity of DDA-MS compared to MRM-MS. The three early detection biomarkers CO9, ITIH3 and A2MG interacted in a tight network (enrichment p-value 3.91e-06) (Figure S4C), which was functionally enriched in the KEGG pathway Complement and coagulation cascades (FDR 0.00183) and in the Uniprot annotated keyword of Serine protease inhibitors (FDR 0.0351, Table S8).

## 4 Discussion

This study, involving multiple independent clinical and pre-clinical sample sets, provides evidence of glycoproteins as serum biomarkers for HGSOC and lays the foundation for further research on larger patient cohorts. Excitingly, three glycoproteins (CO9, ITIH3 and A2MG) were altered in pre-clinical serum samples collected 11.1±5.1 months prior to HGSOC diagnosis, suggesting promise in early detection. The observed changes in proteins pulled down by the three lectins (AAL, SNA and STL) suggest alterations in α-fucose, sialic acid and N-acetylglucosamine during HGSOC development that require further characterization.

To increase likelihood of successful biomarker development, our glycoprotein-focused biomarker pipeline addressed the issue of technical and biological variations by using LeMBA as a common platform across all phases, and developing multi-marker panels, respectively. The LeMBA-MRM platform also has the advantage of being able to be deployed as a clinical assay [24] reducing the time it takes for the findings to be translated for patient use. Alternatively, lectin-immunoassays can be developed for the discovered biomarkers [25].

We employed a phased biomarker study design to operate within budget [26] where the discovery phase screen uses a relatively small sample size to generate a shortlist of lectins and protein candidates for validation in a larger cohort. In view of the small discovery samples size, our choice of low-stringency statistics on the discovery data, and experimental design to analyze all candidate proteins against the three selected lectins (using the single MRM assay) was ultimately critical for successful biomarker validation. Notably, only one specific lectin-protein discovery phase candidate (STL-PON1, Table 1) was ultimately confirmed in the validation cohort (Table 2). The validated biomarkers were comprised mostly of lectin-protein combinations that were just outside the initial cut-off for the discovery analysis but were analyzed in the validation cohort as the custom MRM assay was used on all three selected lectins. This outcome highlights the need for data-specific statistical approaches and considered (non-)use of multiple-testing adjustment in discovery science. Aside from statistics and sample size, biological variation related to evolution of the cancer between the discovery clinical (UKOPS) and validation clinical (AOCS) samples is likely to have contributed to the observed differences.

Strikingly, the validated biomarker proteins physically interact and are functionally enriched in the complement and coagulation cascades, two components of the innate immune system. This finding is in line with the higher risk of venous thromboembolism (VTE) in cancer patients [27, 28] and the emerging concept of a tumor “coagulome”, a cancer-driven network of molecular effectors favoring thrombosis or bleeding [29, 30]. The analysis of the cancer coagulome found expression of genes encoding six pro-coagulant and fibrinolytic factors *(F3, PLAU, PLAT, PLAUR, SERPINB2,* and *SERPINE1)* in The Cancer Genome Atlas [29], and correlated with VTE incidence in 32 cancer types in a previously reported Dutch study [27]. While a moderate correlation was found between VTE risk and expression of Tissue Factor (F3), a major pro-coagulant factor [29], the authors noted heterogeneity across tumor types. Intriguingly, while ovarian cancer had one of the highest VTE incidences in this study, the six examined genes showed moderate expression levels in this cancer [29], indicating important roles of other mechanisms and procoagulant factors. Indeed, our findings identified altered glycosylation of two additional proteins not yet considered in the cancer coagulome, namely thrombin (THRB) and coagulation factor X (FA10, Table 3), which should be evaluated for inclusion in the coagulome.

Proteins of the complement and coagulation cascades interact at several levels, share common regulator proteins and both systems act on immune and endothelial cells [31, 32]. Interestingly, both cascades are activated or regulated by extracellular vesicles [30, 32], small membrane-enclosed vesicles released by cells for inter-cellular communication. Our biomarkers were enriched in the Gene Ontology term “blood microparticles”, defined as a type of extracellular vesicle (EV) devoid of nucleic acids, released from several cell types including platelets, blood cells and endothelial cells. This finding is supported by two previous independent cohort studies reporting elevated serum/plasma EV procoagulant activity in ovarian cancer patients [33, 34]. While a recent meta-analysis reports VTE to be higher in advanced serous ovarian cancer, clear cell histology and ascites at diagnosis [35], our study has found evidence of perturbations in the glycosylation of complement and coagulation cascade several months prior to ovarian cancer diagnosis.

We were highly encouraged to find three validated biomarkers to be altered several months prior to cancer diagnosis in the UKCTOCS set. Levels of two candidates were elevated (glycoforms of C9 and ITIH3) and one was lower (SNA-A2MG) compared to healthy and benign samples. All three proteins are associated with inflammation, with C9 and A2MG having known roles in the complement and coagulation pathways. Of the three potential early detection biomarkers, only A2MG has previously been reported to be altered in ovarian cancer. Similar to our findings, Miyamoto et al. reported decreased A2MG protein in ovarian cancer patients compared to healthy controls [36]. A2MG is a broad-spectrum protease inhibitor which inhibits thrombin and the complement pathway [37]. Reduced A2MG levels may indicate elevated coagulation and complement pathway activity, although the role of the SNA-A2MG glycoform is currently unknown.

C9 is the terminal component of the complement cascade, which has also been found to be elevated in serum of gastric [38], lung [39], colorectal [40, 41] and esophageal cancers [17, 18] through proteomics or glycoproteomic approaches. In our esophageal adenocarcinoma biomarker study, C9 glycoforms binding to each of the 6 short-listed lectins (AAL, EPHA, JAC, NPL, PSA, WGA) was also significantly elevated in esophageal adenocarcinoma compared to the precursor benign condition, Barrett’s esophagus but with some variability in healthy groups [18]. The current study shortlisted three lectins (AAL, SNA, STL) for the validation phase, and all three C9 glycoforms measured were significantly elevated in HGSOC, suggesting that C9 is subjected to increased aberrant glycosylation that may contribute to disease progression. Interestingly, AAL-C9 glycoform levels have been reported to be elevated in lung and stomach cancers, intermediate in hepatocellular carcinoma and low in breast cancer [39]. Recently, we reported the release of C9^+^ EVs by esophageal adenocarcinoma cells as a potential mechanism of the elevated serum C9 glycoform in esophageal cancer [42]. However, the specific glycosylation differences in cancer serum and EVs, as well as the molecular mechanisms underpinning the altered C9 glycosylation in different cancer types remains to be determined.

Inter-alpha-trypsin inhibitor family members, including ITIH3, have been implicated in inflammation and carcinogenesis [43]. In addition to its protease inhibitor activity, ITIH3 is thought to stabilize the extracellular matrix through binding to hyaluronic acid [44].

Interestingly, previous reports suggest blood ITIH3 to be elevated for a similar range of cancers as C9, namely, lung [45], gastric [46], pancreatic [47] and colorectal [48] cancers.

In conclusion, we report the discovery and validation of serum glycoprotein markers for HGSOC using a lectin-assisted proteomics workflow that is directly translatable to blood tests. The validated markers show high specificity when bench-marked against the existing ovarian cancer biomarker, CA125. Their utility in ovarian cancer diagnosis and monitoring will need to be evaluated in additional cohorts, such as at diagnosis and following surgery/chemotherapy. Furthermore, several markers were elevated months prior to cancer diagnosis, and should be further evaluated as for ovarian cancer screening. Functional enrichment of the validated markers highlights blood microparticle (EV)-mediated complement and coagulation activity ahead of clinical diagnosis of HGSOC. Further investigation on the contribution of EV-mediated complement and coagulation in ovarian cancer development may provide mechanisms for prevention.

## 5 Associated Data

The mass spectrometry proteomics data for the discovery phase have been deposited to the ProteomeXchange Consortium via the PRIDE [49] partner repository with the dataset identifier **PXD032299.** The targeted mass spectrometry data for the validation phase have been deposited to PRIDE via Panorama Public database with the dataset identifier **PXD033108.**

## Supplementary Material

Supplementary Material

Table S4

Table S5

Table S6

Table S7

Table S8

Table S9

## Figures and Tables

**Table 1:** Discovery phase biomarker candidates. For each lectin, significant proteins with p-value <0.05 and log_2_FC >1 identified in each clinical cohort have been detailed out below. The total number of candidate proteins across each lectin and clinical cohort accounts for overlaps. The proteins are labelled by their UniProt entry name.

	HGSOC vs Benign		HGSOC vs Healthy		TOTAL NUMBER
**LECTIN**	**UKOPS**	**UKCTOCS**	**UKOPS**	**UKCTOCS**	
**AAL**	A1AG1, A1AG2, FA5, HGFL	MASP1	SEPP1	MASP1	**6**
**CON-A**	FIBB, FCN3	-	HV551	APOM	**4**
**ECA**	LUM, LG3BP	APOH, CO7, CO8G, FBLN1, PON1	CO9, LG3BP, CO8B, CO8G, LUM, THBG	CO8G, FBLN1, PGRP2	**11**
**L-PHA**	PON1, CERU, ITIH3	-	-	-	**6**
**SNA**	FA10, CO8G, FIBA	KAIN, F13B, ZA2G	IGHG2, CBPN, FA10	-	**9**
**STL**	PON1, FIBA, FIBB	LG3BP	CD5L, CHLE, PON1, FA10, ACTG, CBG	A1AT, ITIH3, HEP2, IGLL5, LG3BP	**13**
**WFA**	GELS	A1AT, CO9, ALDOA, HV307	-	IGLL5, CO7, FA12	**8**
**TOTAL NUMBER**	**15**	**15**	**14**	**12**	

**Table 2:** Validated biomarker glycoproteins. Tables shows the number of significant peptides for either HGSOC vs benign or HGSOC vs healthy comparison (q-value <0.05 and log_2_FC >0.5), the number of proteins with any significant peptide, and the number of proteins with all measured peptides significant and consistent in direction. Protein with all peptides consistent are arranged by alphabetical order of their Uniprot entry name, according to the direction of change in HGSOC.

	AAL	SNA	STL
Number of significant peptides	47	41	38
Protein numbers with any significant peptide	23	20	19
Protein numbers with all peptides consistent	5 (21.7%)	6 (30%)	6 (31.6%)
Increased in HGSOC	A1AT, AACT, CO9, HPT	AACT, CO9, ITIH3	CO9, ITIH3
Reduced in HGSOC	A2MG	A2MG, ALS, IBP3	A2MG, ALS, IBP3, PON1

**Table 3:** Signature peptides from lectin-pulldowns for distinguishing HGSOC from benign/healthy and the stability of each peptide assessed by number of times selected during cross-validations.

Lectin (sample size)	AAL (n=70)	SNA (n=95)	STL (n=95)	Combined (n=95)
**Model performance (range)**	AUC = 87.6% (78.4%, 96.8%)	AUC = 84.6% (76.1%, 93.0%)	AUC = 85.0% (76.6%, 93.4%)	AUC = 84.8% (76.4%, 93.3%)
	Sens = 70.4% (51.5%, 84.1%)	Sens = 64.1% (48.4%, 77.3%)	Sens = 64.1% (48.4%, 77.3%)	Sens = 64.1% (48.4%, 77.3%)
	Spec = 90.7% (78.4%, 96.3%)	Spec = 87.5% (76.4%, 93.8%)	Spec = 85.7% (74.3%, 92.6%)	Spec = 89.3% (78.5%, 95.0%)
**UniProt Name_Peptide**	**Times selected**	**Times selected**	**Times selected**	**Times selected**
**IBP3_ALAQCAPPPAVCAELVR**	70 (100%)	95 (100%)	95 (100%)	SNA 95 (100%) STL 87 (91.6%)
**A1AT_QINDYVEK**	70 (100%)			
**HPT_VTSIQDWVQK**	69 (98.5%)		83 (87.4%)	
**IBP3_FLNVLSPR**	69 (98.5%)			
**GELS_QTQVSVLPEGGETPLFK**	68 (97.1%)			
**FETUA_HTLNQIDEVK**	64 (91.4%)			
**FIBB_YQISVNK**	46 (65.7%)			
**FA10_ETYDFDIAVLR**		94 (98.9%)		
**A2MG_NEDSLVFVQTDK**		93 (97.9%)	95 (100%)	STL 95 (100%)
**CHLE_NIAAFGGNPK**		93 (97.9%)	67 (70.5%)	SNA 92 (96.8%)
**KNG1_ENFLFLTPDCK**		93 (97.9%)		
**ITIH2_VQSTITSR**		90 (94.7%)		
**ITIH3_EHLVQATPENLQEAR**		86 (90.5%)	91 (95.8%)	
**C1QC_QTHQPPAPNSLIR**		85 (89.5%)		
**HPT_DYAEVGR**		84 (88.4%)		
**THRB_SGIECQLWR**		79 (83.2%)		
**KNG1_EGDCPVQSGK**			95 (100%)	STL 95 (100%)
**FIBB_QDGSVDFGR**			78 (82.1%)	
**FA10_IVGGQECK**				STL 66 (69.5%)
**FIBB_SILENLR**				STL 60 (63.2%)

AUC, area under the receiver operating curve; Sens, sensitivity; Spec, specificity.

## Statement of clinical relevance

Ovarian cancer continues to be associated with high disease mortality. Much of this is due to the diagnosis at an advanced stage of the most common and aggressive histotype - high grade serous ovarian cancer (HGSOC). This has led to significant efforts to detect the disease earlier when treatment is more effective. However, to date there is no effective screening test. We describe discovery and validation of a novel blood glycoprotein signature using lectin magnetic bead array (LeMBA)-coupled mass spectrometry for HGSOC using a case control study of clinical samples collected at diagnosis. Three biomarker candidates were altered 4-18 months prior to cancer diagnosis in a pilot case control study using pre-clinical samples from the UK Collaborative Trial of Ovarian Cancer Screening. Our findings suggest that serum glycoproteins could be novel biomarkers for earlier detection of HGSOC and lay the foundation for further study in larger cohorts.

## List of Abbreviations

AAL: Aleuria Aurantia Lectin
AOCS: Australian Ovarian Cancer Study
Con-A: Concanavalin-A
ECA: Erythrina Cristagalli Lectin
HGSOC: High grade serous ovarian cancer
LeMBA: Lectin magnetic bead array
PHA-L: Phaseolus Vulgaris Leucoagglutinin
SNA: Sambucus Nigra Lectin
STL: Solanum Tuberosum Lectin
WFA: Wisteria Floribunda Lectin
UKCTOCS: United Kingdom Collaborative Trial of Ovarian Cancer Screening
UKOPS: United Kingdom Ovarian Population Study
