# Supplementary material for: Discovery and validation of serum glycoprotein biomarkers for high grade serous ovarian cancer
Source: Proteomics Clin Appl. 2023 Jun 1;17(4):2200114. doi: 10.1002/prca.202200114 (PMC7615076; doi:10.1002/prca.202200114)
Supplement: Supplementary file 1 — Supplementary information [file PRCA-17-2200114-s004.pdf]

Supporting information for

Discovery and validation of serum glycoprotein biomarker signature for high grade serous ovarian cancer

Mriga Dutt[1], Gunter Hartel[1], Renee S. Richards[1], Alok K. Shah[1], Ahmed Mohamed[1], Sophia Apostolidou[2], Aleksandra Gentry-Maharaj[2], Australian Ovarian Cancer Study Group, John D. Hooper[3], Lewis C. Perrin[3,4], Usha Menon[2]*, Michelle M Hill[1,5]*

Figure S1. Quality control of validation LeMBA-LC-MRM-MS data using internal standard.

Figure S2. Quality control of validation LeMBA-LC-MRM-MS data using SIS peptides.

Figure S3. Normal distribution of validation LeMBA-LC-MRM-MS data

Figure S4. Protein-protein interaction network for the validated biomarkers

Table S1. Clinical information for the discovery phase UKOPS and UKCTOCS cohorts

Table S2. Australian Ovarian Cancer Study (AOCS) cohort information

Table S3. Coefficient of Variation (%CV) of the peptide standards for the LeMBA-MRM-MS cohort.

Table S4 (separate excel file). UKOPS cohort LeMBA-MS data and statistics

Table S5 (separate excel file). UKCTOCS cohort LeMBA-MS data and statistics

Table S6(separate excel file). AOCS cohort LeMBA-MRM-MS data

Table S7 (separate excel file). Univariate statistics for biomarker validation data

Table S8 (separate excel file). Functional enrichment analysis of biomarker proteins

Table S9 (separate excel file). Multivariate statistics for biomarker signatures

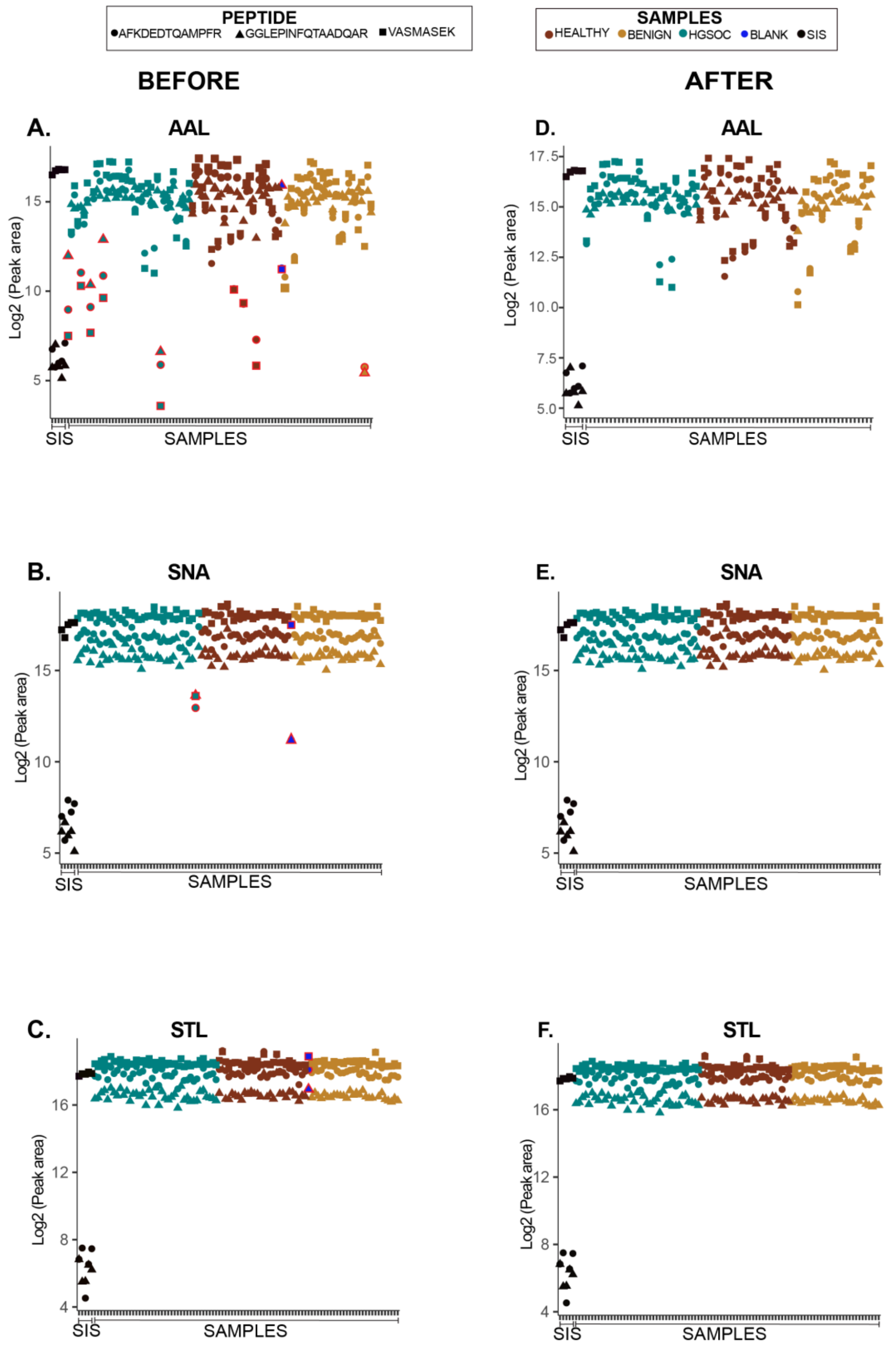

Figure S1. Quality control of validation LeMBA-LC-MRM-MS data using internal standard. Log2 transformed mass spectrometry peak areas for the three measured chick ovalbumin peptides for (A,D**)** AAL, (B, E) SNA and (C, F**)** STL have been presented. The peak area distribution for all samples in a mass spectrometry run have been shown (A, B, C) before and (D, E, F**)** after outlier removal. Outliers have been indicated in red outline.

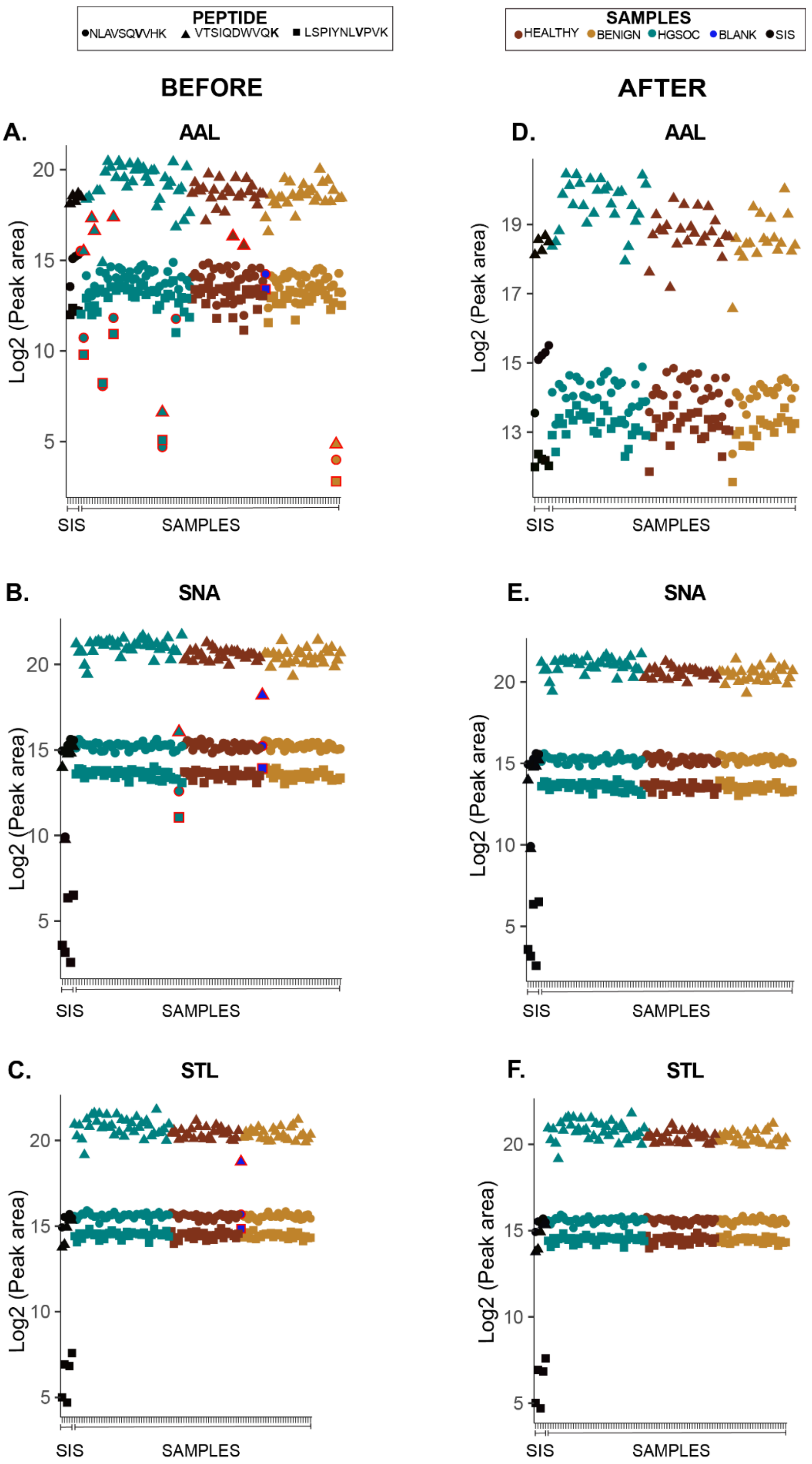

Figure S2: Quality control of validation LeMBA-LC-MRM-MS data using SIS peptides. Log2 transformed mass spectrometry peak areas for the three measured SIS peptides for (A,D**)** AAL, (B, E) SNA and (C, F**)** STL have been presented. The peak area distribution for all samples in a mass spectrometry run have been shown (A, B, C) before and (D, E, F**)** after outlier removal. Outliers have been indicated in red outline.

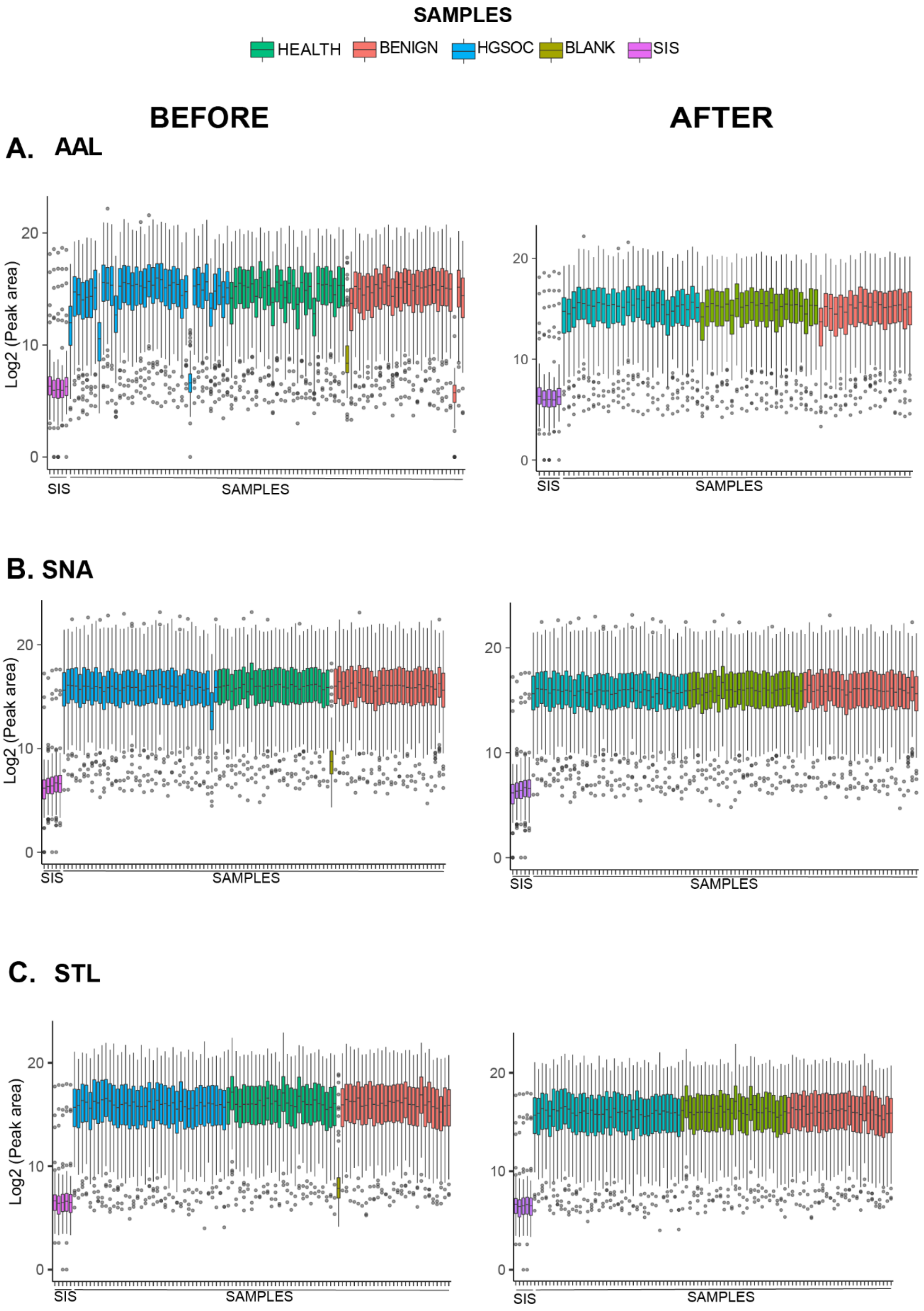

Figure S3. Normal distribution of LeMBA-LC-MRM-MS data. Distribution of log2 transformed mass spectrometry of peak areas for (A) AAL, (B) SNA and (C) STL pulldowns, before and after removal of outliers.

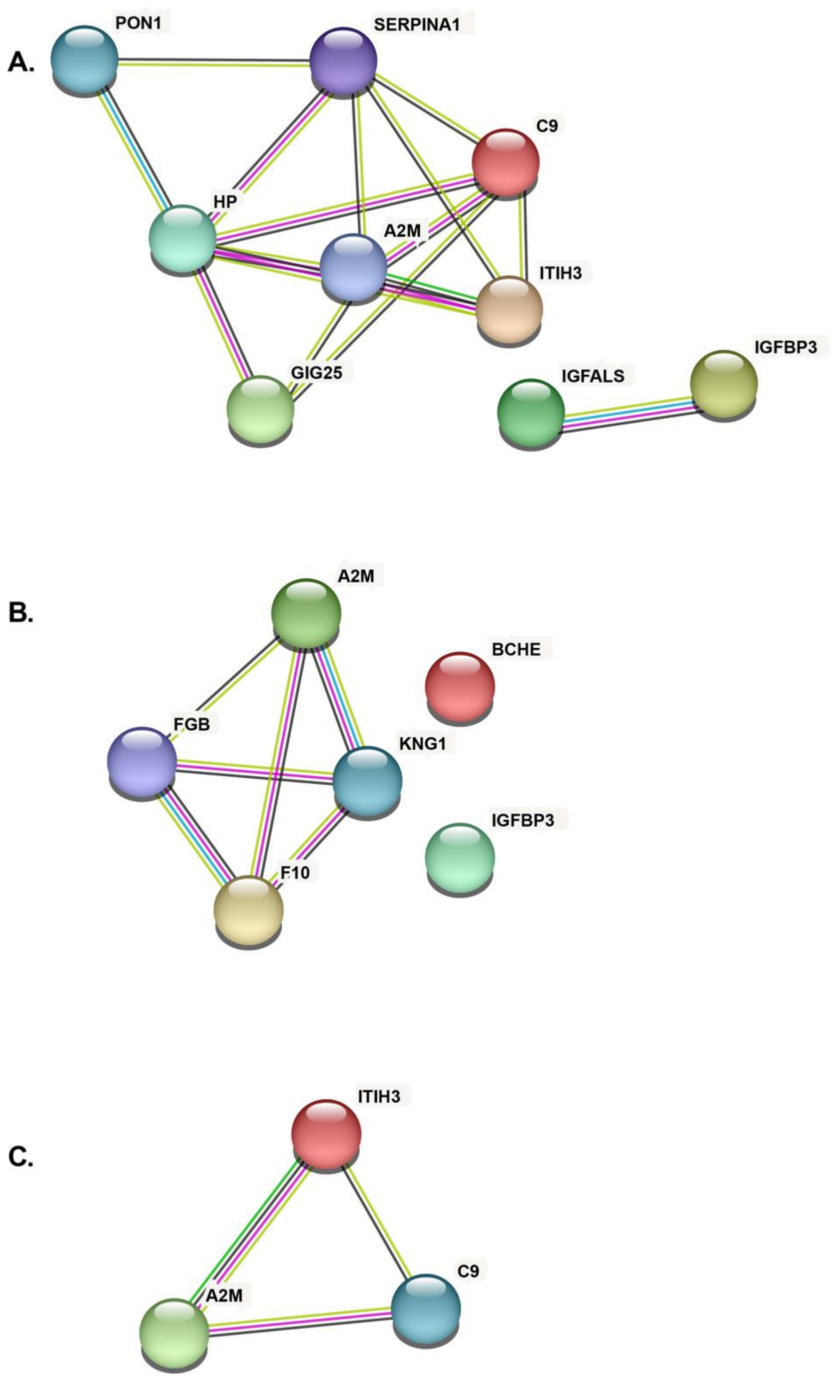

Figure S4. Protein-protein interaction network for the validated biomarkers. STRING database was used to develop protein-protein interaction network of (A) the 9 validated glycoprotein biomarkers from univariate analysis, (B) the 6 glycoprotein biomarkers from the multivariate signature for HGSOC vs (benign or healthy) and (C) the three early HGSOC biomarkers. Nodes are the glycoprotein biomarkers labelled by GeneName. Edges denote interactions between proteins.

Table S1: Clinical information for the discovery phase cohorts.

	UKOPS			UKCTOCS		
	**Healthy**	**Benign**	**HGSOC**	**Healthy**	**Benign**	**HGSOC**
**Samples**	10	10	10	10	10	10
**Age[a] (median ± SD)**	59.5 ± 5.9	59.4 ± 6.1	59.9 ± 6.3	65.2 ± 6.1	65.7 ± 5.9	65.0 ± 6.0
**BMI[b] (median ± SD)**	25.4 ± 4.0	24.9 ± 3.0	24.8 ± 3.5	25.6 ± 3.0	24.9 ± 2.9	28.6 ± 5.0
**Smoking history[c]**						
Current	1 (10%)	0 (0%)	4 (40%)	1 (10%)	2 (20%)	
Former	1 (10%)	3 (30%)	1 (10%)			
Never	6 (60%)	4 (40%)	3 (30%)	7 (70%)	4 (40%)	1 (10%)
Don't know	2 (20%)	3 (30%)	2 (20%)	2 (20%)	2 (20%)	9 (90%)
**Alcohol consumption[c]**						
Current	6 (60%)	6 (60%)	7 (70%)	5 (50%)	4 (40%)	
Not current	2 (20%)	0 (0%)	1 (10%)	1 (10%)	2 (20%)	1 (10%)
Don't know	2 (20%)	4 (40%)	2 (20%)	4 (40%)	2 (20%)	9 (90%)
**CA125 level (median ± SD)**	9.1 ± 3.3	18.5 ± 67.0	687.1 ± 853.7	14.1 ± 1.8	10.1 ± 3.0	55.7 ± 106.5
**Cancer stage**	n/a	n/a		n/a	n/a	
Stage I			2 (20%)			0
Stage II			0			0
Stage III			8 (80%)			8 (80%)
Stage IV			0			2 (20%)

[a] Age at time of serum collection
[b] Body mass index (BMI) at recruitment
[c] For UKCTOCS, self-reported data at 3.5 years post-randomisation
n/a not applicable

Table S2: Australian Ovarian Cancer Study (AOCS) cohort information. All information was provided at time of study recruitment. BMI, body mass index.

Clinical Parameters	HEALTHY	BENIGN	HGSOC	p-value[a]
**Samples**	28	28	39	
**Age (median ± SD)**	61.0 ± 10.8	60.0 ±10.4	59.0 ± 10.4	
**Age at last menopause (median ± SD)**	(-)	45 ± 7.5	49.5 ± 6.3	
**BMI (median ± SD)**	(-)	26.2 ± 7.7	28.4 ± 6.9	0.5024 (ns)
**Hysterectomy**<br>Yes<br>No	(-)	 9 (32.1%)<br>19 (67.9%)	 8 (20.5%)<br>31 (79.5%)	
**Age at hysterectomy (Median ± SD)**	(-)	34.0 ± 4.5	44.0 ± 10.6	0.0196 (*)
**Smoking history**<br>Current<br>Former<br>Never<br>Don't know	(-)	(-)	 6 (15.4%)<br>11 (28.2%)<br>21 (53.8%)<br>1 (2.6%)	
**CA125 level (median ± SD)**	(-)	20.0 ± 68.8	582.0 ± 1960.8	0.0009 (****)
**Cancer stage**<br>Stage II<br>Stage III	n/a	n/a	 6 (15.3%)<br>33 (84.6%)	

[a] Students T-test; (ns) non-significant; (-) data not available

Table S3: Coefficient of Variation (%CV) of the peptide standards for the LeMBA-MRM-MS cohort

Protein	Peptide	RT[a] (min)	AAL[b] (%)	SNA (%)	STL (%)
Chick Ovalbumin	AFKDEDTQAMPFR	10.78	9.19382	2.57322	2.12455
	GGLEPINFQTAADQAR	13.95	2.78279	2.71137	2.17614
	VASMASEK	3.43	11.20731	1.08562	0.96807
Alpha-1-antichymotrypsin	NLAVSQ**V**VHK	8.41	3.03263	1.27633	0.95917
Haptoglobin	VTSIQDWVQ**K**	12.4	4.22573	2.38945	2.28131
Complement component C9	LSPIYNL**V**PVK	16.94	3.97742	2.74893	2.84605

[a]Retention time (RT); [b]Values for AAL cohort reported after sample removal.
